# Development and validation of a multiplexed targeted HILIC-HRMS assay for quantitative analysis of hepatocellular carcinoma circulating biomarkers

**DOI:** 10.1007/s00216-026-06568-1

**Published:** 2026-05-23

**Authors:** Danila La Gioia, Vicky Caponigro, Anna Lucia Tornesello, Emanuela Salviati, Antonio Malinconico, Fabrizio Merciai, Luigi Buonaguro, Franco M. Buonaguro, Pietro Campiglia, Maria Lina Tornesello, Eduardo Sommella

**Affiliations:** 1 Department of Pharmacy, University of Salerno, Via Giovanni Paolo II 132, Fisciano, SA Italy; 2 Innovative Immunological Models Unit, Istituto Nazionale Tumori IRCCS “Fondazione G. Pascale”, 80131 Naples, Italy; 3 Molecular Biology and Viral Oncology Unit, Istituto Nazionale Tumori IRCCS “Fondazione G. Pascale”, 80131 Naples, Italy; 4 National PhD Program in “RNA Therapeutics and Gene Therapy”, Naples, Italy

**Keywords:** Hepatocellular carcinoma, HILIC, Liquid biopsy, Metabolomics, Targeted, Quantitative

## Abstract

Early and accurate diagnosis of hepatocellular carcinoma (HCC) remains a major clinical challenge, particularly in patients with chronic hepatitis C virus (HCV) infection, where standard biomarkers such as alpha-fetoprotein (AFP) often lack sensitivity and specificity. Numerous biomarkers have emerged from untargeted metabolomics and lipidomics approaches; nevertheless, most of these studies provide only relative amounts and do not perform quantitative analysis as further validation. To address this challenge, we developed and validated a targeted method to quantitatively measure panels of biomarkers previously identified as predictive of HCC in untargeted analyses. A high-throughput (6 min) HILIC-HRMS method, based on a multiplexing-HRMS strategy, was developed and validated to measure a panel of lysophosphatidylcholines (LPCs) and carnitines (CARs) in plasma samples from 92 HCC patients. The method showed satisfactory performances in terms of sensitivity (LOD_avg_: 0.0558 ng/mL, LOQ_avg_: 0.667 ng/mL), accuracy (98.75%), linearity (*R*^2^ = 0.999), and stability (intra-day CV: 0.39%, inter-day CV: 1.46%), moderate matrix effects (14.8%), and recovery (95.82%). Despite analyzing a substantially reduced number of variables with respect to the untargeted approach (24 vs 280), the targeted panel confirmed the diagnostic value of the previously identified signature and remains superior to the AFP classification. Among the measured compounds, LPC 14:0 and LPC 18:2 showed the highest discriminative power (AUC = 0.978, AUC = 0.967). The developed targeted approach demonstrated a promising potential tool for HCC discrimination from other liver diseases, and, by obtaining quantitative values of circulating analytes, could serve as a liquid biopsy approach for early diagnostics and therapy evaluation.

**Graphical abstract:**

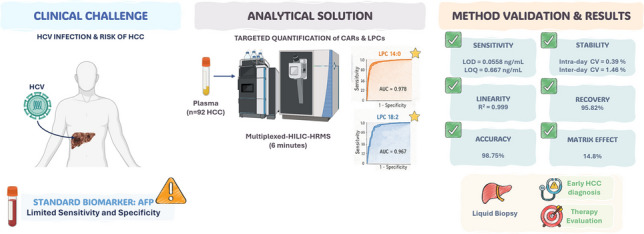

**Supplementary Information:**

The online version contains supplementary material available at 10.1007/s00216-026-06568-1.

## Introduction

Hepatocellular carcinoma (HCC), comprising approximately 75–85% of all primary liver cancers, represents a global health problem with a progressively increasing incidence [1, 2]. The major risk factors for HCC development are hepatitis B (HBV) and C (HCV) virus infection, excessive alcohol consumption, and metabolic dysfunction‐associated steatotic liver disease (MASLD) [3]. In addition, HCC is one of the leading causes of cancer-related mortality worldwide, mainly due to late diagnosis and limited treatment options in advanced stages [4]. Therefore, the development of clinically useful biomarkers for hepatocellular carcinoma (HCC) diagnosis remains a major unmet clinical need. Although α-fetoprotein (AFP) remains the most widely used serum biomarker in clinical practice, its limited sensitivity and specificity, particularly in early-stage disease, severely restrict its diagnostic utility [5]. Consequently, there is an urgent demand for alternative or complementary biomarkers capable of improving HCC early detection and patient stratification. In recent years, plasma cell-free DNA (cfDNA) and circulating tumor DNA (ctDNA) have emerged as promising non-invasive liquid biopsy approaches for early diagnosis, minimal residual disease assessment, and recurrence monitoring in HCC [6, 7]. However, while these strategies offer valuable genomic information, they may not fully capture the downstream functional consequences of tumor-associated metabolic reprogramming. In this context, metabolomics and lipidomics have gained increasing attention as powerful tools for the discovery of circulating biomarkers across multiple diseases, owing to their ability to reflect dynamic biochemical alterations associated with pathophysiological states [8]. Several liquid chromatography–mass spectrometry (LC–MS)-based lipidomics and metabolomics studies, including untargeted, targeted, or pseudotargeted approaches, have reported circulating molecular signatures associated with HCC [9–11]. These investigations consistently highlighted a complex metabolic dysregulation involving bile acids, glycerophospholipids, and the acylcarnitine (CAR) pathways. In this context, through a previous integrated untargeted metabolomics and lipidomics study, we identified a circulating molecular signature that accurately discriminated against HCC patients from chronically HCV-infected subjects, with LPCs and CARs emerging among the most significant discriminatory metabolites outperforming AFP in terms of predictive accuracy [12]. While highly valuable for discovery, relative measurements are not sufficient for clinical implementation, relying on normalized peak areas rather than absolute metabolite concentrations, and thus robust quantification is needed to define reference thresholds and enable inter-study comparability and support longitudinal monitoring [13, 14]. Translation of metabolomics and lipidomics-derived biomarkers into clinically actionable assays, therefore, requires analytically validated targeted methods capable of providing accurate and reproducible quantitative data in complex biological matrices. Given the distinct chemical properties of LPCs and CARs, the development of a single analytical approach capable of their simultaneous quantification remains challenging. Hydrophilic interaction chromatography (HILIC) has emerged as a powerful approach in metabolomics and lipidomics due to its orthogonality to reversed-phase separations, enabling retention of polar metabolites while exploiting polar head-group interactions for lipid separation. This allows co-elution with respective stable isotope-labeled internal standards, ensuring optimal compensation for matrix effects and improving quantitative accuracy [15, 16]. However, most HILIC-based metabolomics and lipidomics methods require dedicated chromatographic conditions and extensive LC–MS optimization [17], frequently resulting in separate targeted assays for polar metabolites [18] or lipid classes [19, 20], respectively. Furthermore, targeted methods for these analyte classes are typically performed in selected/multiple reaction monitoring mode (SRM/MRM) on triple quadrupole MS-analyzers that provide high sensitivity even for analytes in femtomolar concentrations. Despite their known robustness, these approaches may suffer from specificity issues when target analytes share common fragment ions, such as for LPCs and CARs, potentially leading to isobaric interferences, and requiring careful optimization of precursor and fragments transitions as well as collision energies, resulting in longer method development.

Based on these considerations, with the aim to bridge the untargeted discovery and translate these findings into clinically applicable quantitative data, here we developed a high-throughput HILIC-HRMS approach tailored for the simultaneous extraction and quantitation of LPCs and CARs, in a single run. Unlike conventional SRM approaches, the novelty of this method lies in the implementation of a Multiplexed-targeted-SIM (tSIM-MPX) leveraging high-resolution accurate-mass detection benefits at both precursor and fragment level, providing sensitivity and specificity of targeted assays, together with a 6-min HILIC separation that retains the ability to resolve critical regioisomeric species. The ability of the resulting quantitative panel to discriminate HCC from chronic HCV infection was further evaluated, confirming its high classification accuracy. By moving from discovery-based relative profiling to analytically validated absolute quantification, this work aims to advance a clinically translatable liquid biopsy strategy for HCC detection.

## Material and methods

### Chemicals

LC–MS-grade water (H_2_O), acetonitrile (ACN), methanol (CH_3_OH), dichloromethane (DCM), and additives formic acid (HCOOH), acetic acid (CH_3_COOH), ammonium formate (HCOONH_4_), and ammonium acetate (CH_3_COONH_4_) were all purchased from VWR (Milan, Italy). Deuterated and authentic lipid standards were purchased by Avanti Polar Lipids (Alabaster, AL, USA). Unless stated otherwise, other reagents were all purchased by Merck KGaA (Darmstadt, Germany).

### Sample collection and preparation

Briefly, 92 HCV-positive subjects diagnosed with HCC (*n* = 69) and chronic HCV infection (*n* = 23) were enrolled at Istituto Nazionale Tumori IRCCS Fondazione G. Pascale. Complete data on the patients’ characteristics are reported in supplementary material section S1. Plasma samples were collected prior to any treatment, processed within 2 h, and stored at −80 °C until analysis. Plasma samples were extracted as follows: 30 µL of plasma were thawed on ice, and 300 µL of ice-cold ACN, containing a mix of deuterated standards (C16 Carnitine-d_9_, C8 Carnitine-d_9_, C5 Carnitine-d_9_, LPC 17:0-d_5_, LPC 19:0-d_5_), was added and vortexed for 10 min. Subsequently, samples were incubated at −30 °C for 30 min and centrifuged at 14,680 rpm for 10 min at 4 °C to induce protein precipitation. Samples were injected in randomized order, and blank samples were injected regularly and used to assess carry-over and exclude background signals. Dried samples were dissolved in 50 µL of ACN/H_2_O (80/20 *v/v* %). The study was approved by the Institutional Ethical Committee (prot. 30-OSS/22), and it is in accordance with the principles of the Declaration of Helsinki.

### Instrumentation and LC setup

All analyses were performed on a Thermo Vanquish Flex UHPLC coupled online to a hybrid quadrupole Orbitrap Exploris 120 mass spectrometer (Thermo Fisher Scientific, Bremen, Germany) equipped with a heated electrospray ionization probe (HESI II). The separation was performed with an Acquity UPLC BEH Amide™ (100 × 2.1 mm × 1.7 µm, 130 Å) protected with a VanGuard BEH Amide™ precolumn (5.0 × 2.1 mm; 1.7 µm, 130 Å) (Waters, Milford, MA, USA). The mobile phases were composed of (A): H_2_O/ACN (95:5 *v/v *%) and (B): H_2_O/ACN (5:95 *v/v *%), both with 10 mM CH_3_COONH_4._ The following gradient was used: 0–1 min, 99%B, 7–8 min 30% B, 8.1–11.5 min 99% B. The column temperature was set at 45 °C, and a flow rate of 0.4 mL/min was used. The injection volume was 2 µL.

### High resolution mass spectrometry (HRMS) parameters

MS data acquisition was performed in targeted-SIM-Multiplexing (tSIM-MPX). MS1 scan OT resolution, 30,000; normalized AGC target (100%); maximum injection time, auto. S-Lens RF level, 70; isolation window, 2 Da; SIM Window Mode: Centre Mass. The HESI source parameters were as follows: sheath gas, 40 a.u.; auxiliary gas, 15 a.u.; sweep gas, 0 a.u. Spray voltages were set to 3.3 kV and 3.0 kV for ESI (+) and ESI (-), respectively. Ion transfer tube and vaporizer temperatures were set to 280 °C and 300 °C, respectively. The instrument was externally calibrated daily with FlexMix solution (Thermo Fisher), while at the beginning of every LC run, the internal calibrant was injected (IC run start mode). The tSIM-MPX conditions for the quantification of CARs and LPCs are described in detail in Table 1. MS data were analyzed by FreeStyle v.1.8 SP2. Isotopic overlap correction was calculated as reported previously [21].

**Table 1:** Overview of the tSIM-MPX acquisition parameters, including monitored ions, m/z values, charge, and MPX ID, for the targeted quantification of carnitines (CARs) and lysophosphatidylcholines (LPCs)

Compound	Formula	Centre Mass (m/z)	z	MSX ID
**CAR 16:0**	C_23_H_45_NO_4_	400.3421	1	1
**CAR 16:0-d9**	C_23_H_36_D_9_NO_4_	409.3986	1	1
**CAR 8:0**	C_15_H_29_NO_4_	288.2169	1	1
**CAR 8:0-d9**	C_15_H_20_D_9_NO_4_	297.2734	1	1
**CAR 5:0**	C_12_H_23_NO_4_	246.1700	1	1
**CAR 5:0-d9**	C_12_H_14_D_9_NO_4_	255.2265	1	1
LPC 17:0	C_25_H_52_NO_7_P	510.3554	1	2
LPC 17:0-d5	C_25_H_47_D_5_NO_7_P	515.3868	1	2
LPC 18:1	C_26_H_52_NO_7_P	522.3554	1	2
LPC 19:0-d5	C_27_H_51_D_5_NO_7_P	543.4181	1	2
**CAR 9:0**	C_16_H_31_NO_4_	302.2326	1	1
**CAR 10:1**	C_17_H_31_NO_4_	314.2326	1	1
**CAR 2:0**	C_9_H_17_NO_4_	204.1230	1	1
**CAR 10:0**	C_17_H_33_NO_4_	316.2482	1	1
**CAR 6:0**	C_13_H_26_NO_4_	261.1935	1	1
**CAR 5:1**	C_12_H_21_NO_4_	244.1543	1	1
LPC 18:2	C_26_H_50_NO_7_P	520.3398	1	2
LPC 20:3	C_28_H_52_NO_7_P	546.3554	1	2
LPC 14:0	C_22_H_46_NO_7_P	468.3085	1	2
LPC 18:3	C_26_H_48_NO_7_P	518.3241	1	2
LPC 20:4	C_28_H_50_NO_7_P	544.3398	1	2
LPC 16:0	C_24_H_50_NO_7_P	496.3398	1	2
LPC 18:0	C_26_H_54_NO_7_P	524.3711	1	2

### Method validation

The method validation was performed by assessing system suitability and performance, linearity, sensitivity, precision, accuracy, and matrix effect, according to ICH Guidelines (https://www.ich.org/page/quality-guidelines).

#### System suitability and performance

The system suitability was tested by 5 consecutive injections using a standard mixture of the analytes and internal standards. System performance was assessed by injecting 1 blank (without the analyte and IS) and one lower limit of quantification (LLOQ). System carry-over was investigated by injecting 2 blank samples immediately after a calibration standard at the upper limit of quantification (ULOQ). Carry-over was considered acceptable when the analyte signal in the blanks did not exceed 20% of the mean analyte response at the LLOQ level (Eq. 1). The re-injection reproducibility of extracted samples was verified by reanalyzing an entire batch after storage in the autosampler at 4 °C. The limit of detection (LOD) and limit of quantification (LOQ) were determined using the calibration curve approach, based on the ratio between the standard deviation of the intercept (σ) and the slope of the regression line (S). Specifically, LOD (Eq. 2) and LOQ (Eq. 3) were calculated according to the following equations:

1 $$Carry\;Over\;(\%)=\frac{Peak\;area\;blank}{Peak\;area\;ULOQ}\times100$$

2 $$LOD=3.3 \times \frac{\sigma }{S}$$

3 $$LOQ=10 \times \frac{\sigma }{S}$$

where *σ* represents the standard deviation of the *y*-intercept of the calibration curve, and *S* is the slope.

#### Calibration samples

Stock solutions of each analyte were prepared by accurately weighing the appropriate amount of compound and dissolving it in methanol (MeOH) to obtain a final concentration of 1 mg/mL. Working standard solutions were freshly prepared on the day of analysis by serial dilution of the stock solutions with acetonitrile/water (ACN/H_2_O, 80/20, *v/v* %). From the stock solutions, a set of calibration standards covering the concentration range from 10 µg/mL to 1 ng/mL was prepared. All stock solutions were stored at − 20 °C and protected from light.

#### Linearity

The linearity was assessed using 12 concentration levels. The calibration curves were generated by plotting the peak area ratio of the analytes at each level to the peak area of IS versus its concentration and fitted using a linear regression model. The calculated concentration was used to determine the content of each analyte in each individual calibration standard and considered acceptable if the mean accuracy was within ± 20% of the nominal concentrations for all calibration points.

#### Sensitivity

The LOD and LOQ values were calculated through serial dilutions according to ICH Guidelines. The calculations were performed using the previous equations (Eqs. 2 and 3). The determination of both LOD and LOQ was carried out in triplicate. The standard curve was prepared on each day of the analyses, and the calculated equations were used to determine the concentrations in all samples within the analytical runs.

#### Precision and accuracy

The precision of the method was evaluated by calculating the relative standard deviation (%RSD) of the back-calculated concentrations of calibration standards across triplicate injections. In addition to the calibration curve, method performance was monitored using a pooled quality control (QC) sample consisting of a mix of all target standards analyzed throughout the study to assess intra- and inter-run variability and ensure analytical stability. Intra-day (repeatability) precision was assessed within a single analytical run, while inter-day (reproducibility) precision was determined by analyzing calibration curves and QC mix standard samples prepared on 3 separate days. Precision was considered acceptable when %RSD did not exceed 15% across all concentrations, except at the LLOQ, where deviations up to 20% were tolerated, in accordance with international bioanalytical method validation guidelines (e.g., EMA, FDA) [22, 23].

#### Recovery

Recovery was determined by comparing the analyte peak areas obtained from extracted samples spiked before extraction with those from samples spiked post-extraction at equivalent nominal concentrations. The recovery (%) was calculated as the ratio between the mean peak area of the pre-extraction spiked samples and that of the post-extraction spiked samples, multiplied by 100 (Eq. 4).

4 $$Recovery\;\left(\%\right)=\frac{Peak\;area\;pre-extraction}{Peak\;area\;post-extraction}\times100$$

This evaluation was performed at three concentration levels (low, medium, and high) of spiked standards within the calibration range, in triplicate. Consistent recovery across concentrations and low variability (expressed as %RSD) indicated adequate extraction efficiency and method reproducibility. The internal standard recovery was also monitored to ensure reliable normalization and correction of potential matrix effects or sample loss during preparation.

#### Matrix effect

The matrix effect was evaluated by comparing the calibration curve slopes obtained from standard solutions prepared in pure solvent with those prepared by spiking blank matrix extracts (post-extraction) at equivalent concentrations.

The matrix effect (%) was calculated as reported in Eq. 5:

5 $$Matrix\;effect\;\left(\%\right)=\frac{Slope\;matrix}{Slope\;solvent}\times100$$

Values lower than 100% indicate ion suppression, while values higher than 100% indicate ion enhancement.

### Data analysis

All statistical analyses and graphical representations were performed using MATLAB R2025b (MathWorks, Natick, MA, USA) with in-house scripts and custom chemometric functions. Concentration data were log_10_-transformed when appropriate for univariate visualization. Receiver operating characteristic (ROC) curve analysis was subsequently performed to evaluate the univariate discriminative ability of individual metabolites. For each quantified compound, ROC curves were computed using the corresponding concentration values as continuous predictors and the class label (HCC vs HCV) as the binary response variable, considering HCC as the positive class. The ROC curve represents the relationship between the true positive rate (sensitivity) and the false positive rate (1 − specificity) obtained by varying the decision threshold across the entire range of observed concentrations. The discriminative performance of each metabolite was quantified by the area under the ROC curve (AUC), which provides a threshold-independent measure of classification capability. An AUC value of 0.5 indicates no discriminative power, whereas values approaching 1.0 indicate increasing separation between classes. Before multivariate analysis, the data matrix was autoscaled (mean-centred and scaled to unit variance) [24]. Unsupervised exploration was performed using principal component analysis (PCA) [25]. Score plots were used to evaluate clustering behavior and detect potential outliers. 95% confidence ellipses were calculated in the score space based on Hotelling’s T^2^ statistic. Loading plots were examined to identify metabolites contributing most strongly to the PCs, and the percentage of explained variance was calculated for each component. For supervised modelling, samples were divided into training (80% of the data) and test subsets using the duplex algorithm [26] applied in PCA score space, accounting for 98% of the explained variance, while preserving class distribution. Supervised classification was performed by partial least-squares discriminant analysis (PLS-DA) [27]. Models were trained on the autoscaled training set, and the optimal number of latent variables (LVs) was selected by minimizing classification error using fivefold cross-validation. Model performance was evaluated on the training, cross-validation, and independent test sets in terms of accuracy, sensitivity, and specificity derived from confusion matrices. RAW MS files associated are available at Zenodo (https://zenodo.org/) under the following: 10.5281/zenodo.19352347.

## Results

### Characteristics of the study population

The study population was predominantly male in both the HCC and HCV groups, with HCC patients being significantly older. HCV infection emerged as the main cause of HCC, and most patients presented with single tumor nodules and moderate (G2) differentiation (supplementary section S1). Biochemical analysis showed increased ALT and GGT levels, while AST and AFP were not significantly elevated in the HCC group compared with the group of patients with chronic HCV [12]. Additionally, AFP levels did not correlate with tumor characteristics, highlighting its limited sensitivity and the need for more reliable biomarkers for early HCC detection.

### Optimization of sample preparation for targeted quantification of carnitines and LPCs

**Fig. 1:**
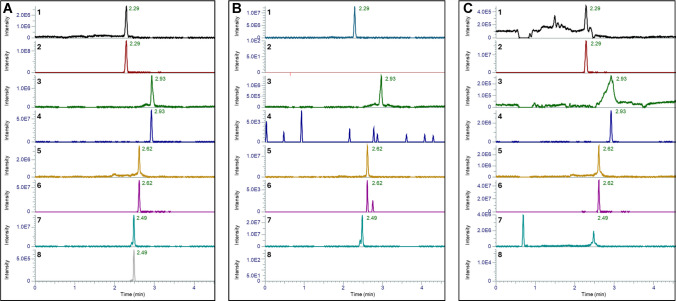
A–C. Extracted ion chromatograms (EICs) of some target metabolites in human plasma obtained using three different sample extraction strategies: (**A**) protein precipitation with ACN 100%, (**B**) protein precipitation with MeOH 80% (*v/v *%), and (**C**) solid-phase extraction (SPE) eluted with MeOH. The comparison highlights differences in signal intensity and chromatographic profiles among the extraction approaches. 1: CAR 16:0, 2: CAR 16:0-d_9_, 3: CAR 5:0, 4: CAR 5:0-d_9_, 5: CAR 8:0, 6: CAR 8:0-d_9_, 7: LPC 17:0, 8: LPC 17.0- d_5_

The selection of the targeted panel was based on the results of our previous untargeted metabolomics and lipidomics study [12], in which acylcarnitines and lysophosphatidylcholines consistently emerged among the most significant discriminatory features. Specifically, these metabolite classes showed high variable importance in projection (VIP) scores in PLS-DA models (VIP > 1.5) and statistically significant differences across groups (one-way ANOVA) (p-value < 0.001*)*, supporting their inclusion in the targeted assay. To maximize recovery of the analytes of interest, different plasma sample-preparation strategies were initially compared, including solid-phase extraction (SPE) and protein precipitation using water-miscible organic solvents. Polymeric reversed phase sorbent was tested, with different wash and elution conditions, SPE workflows exhibited class-dependent selectivity, with variable recoveries across carnitines and LPCs. SPE protocol and recovery values are reported in supplementary material (S2-TabS2). Based on these observations, two different protein precipitation solutions, such as ice-cold ACN (100% *v/v*) and MeOH (80% *v/v*), were compared. The results showed that ACN-based precipitation outperformed the corresponding MeOH-based approach, achieving a mean recovery of 95.82% and 60.21%, respectively, confirming the suitability of ACN-based precipitation for the quantitative recovery of carnitines and LPCs. The improved analytical performance obtained with ACN precipitation was further supported by representative extracted ion chromatograms, reported in Fig. 1.

### Optimization of chromatographic conditions for targeted quantification of carnitines and LPCs

**Fig. 2:**
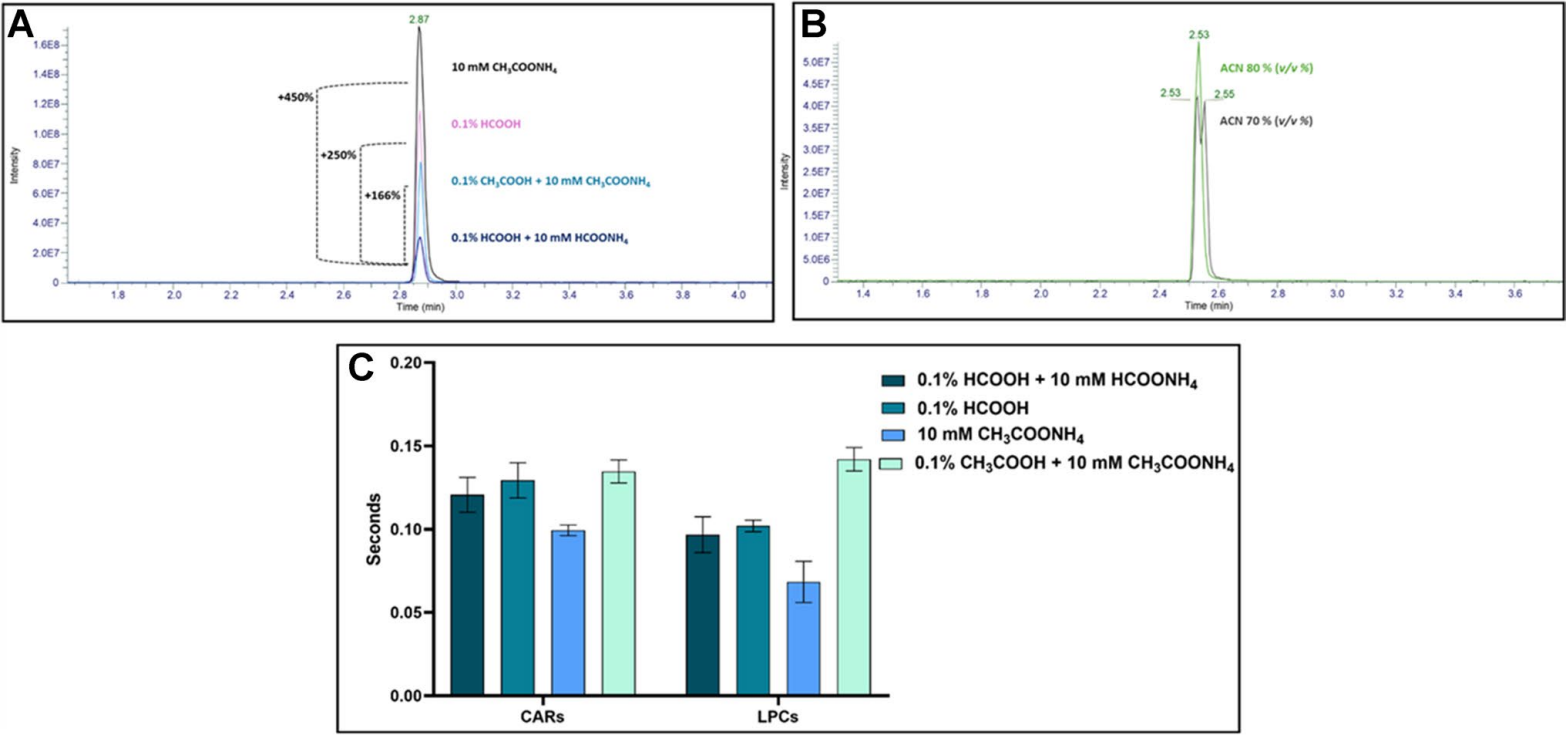
**A** Extracted ion chromatograms (EICs) intensities of CAR C8 under different mobile-phase additives: 10 mM ammonium acetate (AmAc), formic acid (FA), AmAc + acetic acid (AcA), and ammonium formate + FA (AmF + FA). Signal intensity increased by 5.5-fold (450%), 3.5-fold (250%), and 2.67-fold (166.75%) for AmAc, FA, and AmAc + AcA, respectively, relative to AmF + FA. **B** Extracted ion chromatograms (EICs) of CAR 16:0 under optimized solvent conditions, showing improved peak shape and signal intensity at ACN 80% (*v/v* %) (green trace) compared to ACN 70% (*v/v* %) (gray trace). **C** FWHM values of some target metabolites detected in human plasma samples across different mobile-phase modifiers. AmF, ammonium formate; FA, formic acid; AmAC, ammonium acetate; AcA, ammonium acetate

Since the previous untargeted approach was carried out using Hydrophilic Interaction Chromatography (HILIC) separation mode, the stationary phase chemistry was maintained while its geometry was modified using a shorter chromatographic column (150 vs 100 mm), to increase sample throughput. The gradient was accordingly scaled from 14 to 10 min. To maximize the ionization of the CARs and LPCs panel, multiple mobile phase additives based on volatile salts, namely 10 mM of ammonium formate (AmF) or 10 mM ammonium acetate (AmAc), with and without additional acidic modifiers (formic acid (HCOOH) or acetic acid (CH_3_COOH)), were evaluated. Each condition was assessed for its impact on signal-to-noise ratio (*S/N*) and peak symmetry. Among the tested conditions, the mobile phase composed of H_2_O/ACN (95:5, *v/v *%) with 10 mM ammonium acetate as mobile phase A and H_2_O/ACN (5:95, *v/v *%) with 10 mM ammonium acetate as mobile phase B, without additional acidic modifiers, provided the best overall performance, in terms of intensity and peak symmetry among all target analytes compared to other conditions. Signal intensity increased by 5.5-fold (450%), 3.5-fold (250%) and 2.67-fold (166.75%) when using AmAc, FA and AmAc + AcA, respectively, compared to AmF + FA (Fig. 2A).

Further optimization focused on sample resuspension solvent composition, which was adjusted using a higher proportion of organic solvent, specifically from ACN 70% (*v/v *%) to ACN 80% (*v/v *%) (Fig. 2B). This optimization prevented peak splitting of some carnitines and enhanced analyte focusing on the column head.

**Fig. 3:**
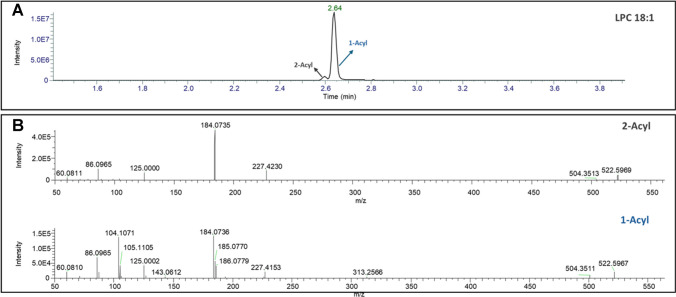
**A** Extracted ion chromatogram (EIC) of **LPC 18:1** under optimized HILIC conditions, showing the separation of LPC regioisomer (sn-1/sn-2). **B** Representative MS/MS spectra displaying diagnostic fragment ions and relative intensity used for regioisomer discrimination

Significantly, the optimized HILIC method allowed the chromatographic separation of LPCs regioisomers (Fig. 3A). Different retention times were recorded for LPC positional isomers that varied in the acyl chain position (*sn*−1/*sn*−2), and this was further supported by their MS/MS spectra (Fig. 3B). Extracted ion chromatograms showed near-baseline resolution, and the relative abundance of diagnostic fragment ions supported the structural assignment of each isomer.

### Selection of HRMS acquisition mode for targeted quantification of carnitines and LPCs

**Fig. 4:**
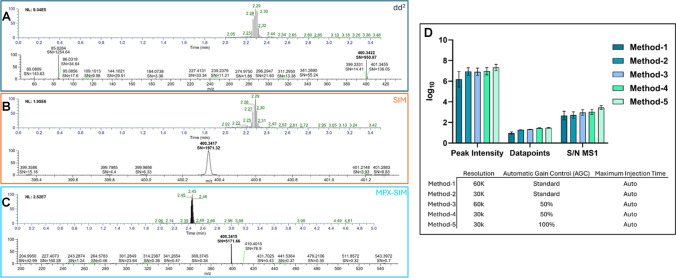
** A**–**D** Comparative assessment of acquisition strategies for trace-level metabolite detection. S/N ratios and peak areas measured under different acquisition modes: **A** DDA, **B** SIM, **C** tSIM-MPX. Representative chromatographic profiles demonstrating enhanced sensitivity and reproducibility with multiplexed SIM. Evaluation of Orbitrap MS1 acquisition parameters for optimized chromatographic sampling in tSIM-MPX

Different High-Resolution Mass Spectrometry (HRMS) acquisition strategies, including Full Scan-Data-Dependent Acquisition (FS-DDA), Selected Ion Monitoring (SIM), and targeted-Selected Ion Monitoring-Multiplexing (tSIM-MPX), were systematically assessed for the targeted assay of carnitines and LPCs in human plasma. Among these, the tSIM-MPX method demonstrated superior analytical performance in terms of chromatographic sampling, sensitivity, and quantitative robustness. Notably, multiplexed tSIM-MPX provided at least 10 data points per chromatographic peak for all monitored analytes, thereby meeting established criteria for robust targeted quantification and outperforming both FS-DDA and SIM acquisition modes which resulted in reduced sampling frequency (Fig. 4A, B). Comparative evaluation of *S/N* and peak areas across acquisition modes demonstrated that tSIM-MPX yielded superior analytical signals for all analytes, with marked improvements in both sensitivity and signal reproducibility, thus being more suitable for low-abundance analytes (Fig. 4C). Additionally, targeted MS/MS experiments were performed to confirm their structural identities by monitoring class-specific diagnostic fragments. LPCs species were confirmed by the characteristic phosphocholine ion m/z 184.0733 (C_5_H_15_NO_4_P^+^), while carnitines were validated by the fragment at 85 m/z (C_4_H_5_O_2_^+^), which is the most significant feature peak of CAR spectra (Fig. S1 A, B). In parallel, different Orbitrap MS1 resolution settings (30,000 and 60,000) were evaluated to balance mass accuracy with scan speed, ultimately selecting the resolution that provided optimal chromatographic sampling. Furthermore, various Automatic Gain Control (AGC) values were assessed (automatic, 50%, and 100%) to ensure ion accumulation of low-abundance species without extending the scan cycle time (Fig. 4D, Tab. S2). Consequently, multiplexed SIM was selected as the acquisition strategy for subsequent targeted analyses.

### Performance evaluation of the optimized SIM-MPX-based LC–HRMS method

The final targeted HILIC–HRMS method was validated according to ICH guidelines, assessing key performance parameters including sensitivity, accuracy, intra- and inter-day precision, matrix effects (ME), carry-over, and extraction recovery. In this context, to improve quantification accuracy and compensate for matrix-induced ionization, the set of deuterated internal standards was selected to match the different chemical properties of the analytes, covering short, medium, and long acyl chain CARs and at least two LPCs species; absolute quantitative measurements were thus obtained by multi-point calibration curves and class-representative isotopically labeled internal standards (Table 2). Validation results confirmed that the optimized workflow met all acceptance criteria, with low variability and minimal matrix interference. In this context, the CARs and LPCs elution window was separated from phosphatidylcholines (PCs) elution region, thus avoiding strong ion suppression (Fig. S2). The combination of tSIM-MPX acquisition, high mass resolution, and optimized MS parameters provided excellent sensitivity and reproducibility, establishing this approach as a robust solution for high-throughput targeted quantification of carnitines and LPCs in complex biological matrices. Furthermore, since more I.S for the LPCs and CARs subclass were used, we applied a cross-quantification approach to evaluate the calculated quantitative error. So, we used the known amount of the first IS to quantify the second IS, and vice versa. By comparing the calculated IS amount against the expected value (theoretical), we obtained an overall average error of 8.5% for LPCs and CARs panel, confirming the reliability of our quantification process.

**Table 2:** Analytical validation of the developed HILIC-tSIM-MPX method, reporting linearity, sensitivity (LOD and LOQ), intra- and inter-day precision, accuracy, repeatability, recovery and matrix effect. *LOD*, limit of detection; *LOQ*, limit of quantification; *ME*, matrix effect; *CV*, coefficient of variation; *RT*, retention times

Metabolite	R^2^	LOD	LOQ	Accuracy	Recovery	ME	Interday		Intraday	
		ng/mL					CV% areas	CV% RT	CV% areas	CV% RT
CAR 16:0-d9	0.997	3.87 ± 1.17	11.73 ± 1.18	98.55% ± 0.01	91.43% ± 0.42	5% ± 0.54	5.89%	0.41%	0.13%	0.00%
CAR 8:0-d9	0.995	0.49 ± 0.15	1.50 ± 0.16	98.77% ± 0.00	106.61% ± 0.33	16% ± 0.48	0.25%	0.36%	0.13%	7.09%
CAR 5:0-d9	0.999	0.08 ± 0.02	0.27 ± 0.03	98.10% ± 0.01	85.34% ± 0.73	13% ± 0.57	15.49%	0.66%	0.12%	0.23%
LPC 17:0-d5	0.999	0.12 ± 0.05	0.36 ± 0.05	98.55% ± 0.00	96.39% ± 0.18	20% ± 1.65	15.53%	0.44%	3.44%	0.00%
LPC 18:1-d5	0.999	0.18 ± 0.03	0.56 ± 0.04	98.65% ± 0.00	94.78% ± 0.26	20% ± 1.42	1.01%	0.59%	0.06%	0.00%

### Differential LPCs and CARs profile in HCC

**Fig. 5:**
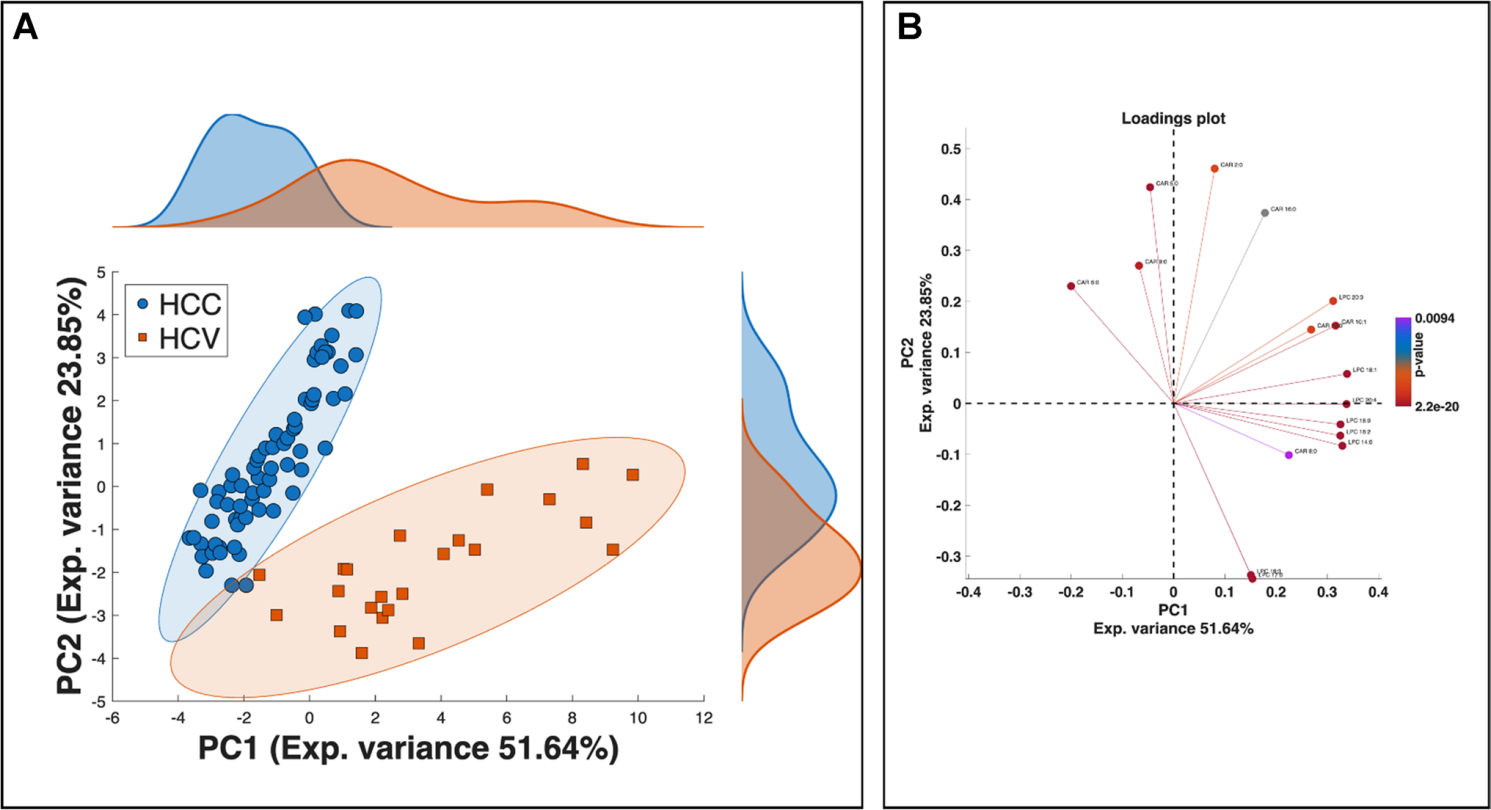
** A** PCA scores plot (PC1 vs PC2) obtained after autoscaling of the concentration data. 95% confidence ellipses were calculated for each class based on Hotelling’s T^2^ statistic in the PCA score space. **B** The loadings plot was derived from the PCA loading matrix and represents the contribution of each metabolite to the first two principal components. HCC, hepatocellular carcinoma; HCV, hepatitis C virus infection

**Fig. 6:**
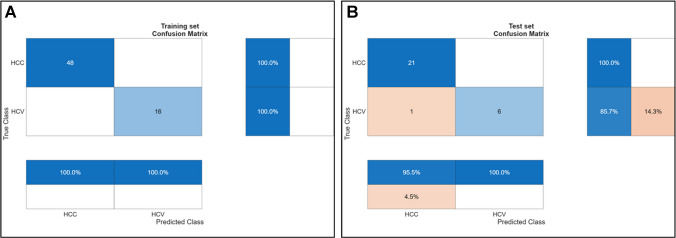
**A**, **B** Supervised classification was performed using partial least-squares discriminant analysis (PLS-DA) coupled with linear discriminant analysis (LDA). The number of latent variables (nLVs = 2) was selected by fivefold cross-validation. Confusion matrices were generated from predicted versus true class memberships for both the **A** training and **B** independent test sets and are displayed with row- and column-normalized percentages

**Fig. 7:**
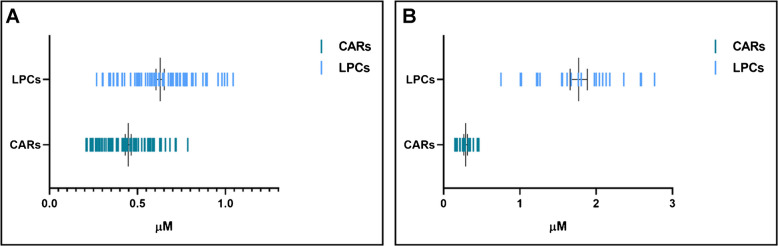
** A**, **B** Aligned line plot of CARs and LPCs quantified in patients with HCC (**A**) and HCV (**B**) patients. Data are presented as mean ± SEM

PCA was applied to the autoscaled concentration data (Fig. 5A, B). PC1 explained 51.64% of the total variance, and PC2 accounted for 23.85%, retaining a cumulative variance of 75.49% for the first two components. The scores plot (Fig. 5A) shows a clear separation between HCC and HCV samples, with minimal overlap. HCC samples were predominantly distributed along the negative side of PC1, whereas HCV samples were mainly located on the positive side, indicating that PC1 represents the main axis of discrimination. Intra-group variability was limited, particularly within the HCC cohort. The corresponding loadings plot (Fig. 5B) highlights the metabolites contributing to this separation and provides a graphical overview of those most strongly associated with class discrimination, with the corresponding p-values reported through the color scale. This representation highlights the most statistically relevant discriminatory molecules, while individual log_10_-transformed concentration distributions for each metabolite are reported in Fig. S3, allowing direct visualization of group-wise differences between HCC and HCV samples. LPCs are predominantly associated with the positive direction of PC1 and are characterized by lower p-values, indicating stronger statistical significance. In contrast, several carnitine species contribute to the opposite direction along PC1. Metabolites positioned at the extremes of the loading vectors had the smallest p-values, confirming their major influence on class discrimination and supporting the differential lipidomic signature observed between HCC and HCV. Supervised classification was subsequently performed using PLS-DA. The optimal model achieved 100.00% accuracy, sensitivity, and specificity in both the training and cross-validation phases. When applied to the independent test set, the model maintained high predictive performance, with an overall accuracy of 96.43%. Sensitivity for HCC was 100%, whereas sensitivity for HCV was 85.71%, due to a single misclassified HCV sample. Specificity values were 85.71% for HCC and 100.00% for HCV. Confusion matrices were generated from predicted versus true class assignments and are displayed with row- and column-normalized percentages (Fig. 6A, B). The performance of the presented model is consistent with the previously reported omics-based PLS-DA results and remains superior to the AFP classification [12]. In the earlier analysis, AFP classification showed limited discriminative power, with an accuracy of 48.98% in the training set and 55.00% in the test set (AFP < 20 ng/mL considered misclassified), confirming its modest effectiveness as a standalone marker for HCC identification, indicating limited discriminative capability with respect to the developed targeted assay. Quantitative analysis of the analyte panel revealed clear stratification between clinical groups (Tab S2, Fig. 7). Scatter plot representations of log_10_-transformed concentrations (Fig. S3) showed statistically significant differences for multiple LPCs and CARs species between HCC and HCV groups. Statistical comparisons were performed using the Mann–Whitney U test followed by FDR correction (*p < 0.05; **p < 0.001). LPCs levels were globally reduced in HCC vs HCV, while CARs showed acyl-chain length differences, and these results agree with previous untargeted analyses.

**Fig. 8:**
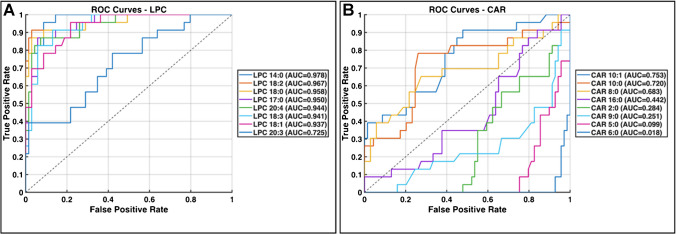
**A**, **B** Receiver operating characteristic (ROC) curves. Each curve represents the univariate classification performance of a single LPC (**A**) and CAR (**B**) metabolite in discriminating HCC from HCV samples. The x-axis indicates the false positive rate, and the y-axis the true positive rate across varying thresholds. The corresponding area under the curve (AUC) values are reported in the legend, highlighting the strong discriminative capability of most LPC species. For metabolites with ROC curves below the diagonal, AUC values below 0.5 reflect an opposite direction of regulation under the selected ROC orientation and should be interpreted together with the corresponding concentration distributions reported in Fig. S3

The discriminatory contribution of the monitored panel was further evaluated by ROC curve analysis (Fig. 8A, B). The ROC curves obtained for LPC species (Fig. 8A) demonstrate a consistently strong classification performance between HCC and HCV samples. Most LPC variables exhibited high area under the curve (AUC) values, confirming their strong diagnostic potential. LPC 14:0 showed the highest discriminative power (AUC = 0.978), followed by LPC 18:2 (AUC = 0.967), LPC 18:0 (AUC = 0.958), and LPC 17:0 (AUC = 0.950). Additional species such as LPC 20:4 (AUC = 0.944), LPC 18:3 (AUC = 0.941), and LPC 18:1 (AUC = 0.937) also demonstrated excellent classification capability. Only LPC 20:3 displayed a comparatively lower performance (AUC = 0.725), although still indicating moderate discriminative ability. The consistently high AUC values across most LPCs corroborate and reinforce the key role of this lipid subclass in differentiating HCC from HCV. In contrast, the ROC analysis of CARs (Fig. 8B) revealed a more heterogeneous diagnostic profile. Medium-chain carnitines showed moderate discriminative capability, with CAR 10:1 (AUC = 0.753), CAR 10:0 (AUC = 0.720), and CAR 8:0 (AUC = 0.683). In the present ROC representation, AUC values above 0.5 correspond to metabolites showing higher concentrations in HCV, whereas metabolites enriched in HCC appear with ROC curves below the diagonal and AUC values below 0.5. Accordingly, several CARs, including CAR 6:0 (AUC = 0.018), CAR 5:0 (AUC = 0.099), CAR 9:0 (AUC = 0.251), and CAR 2:0 (AUC = 0.284), showed curves below the diagonal**.** These values do not indicate an absence of discriminatory information but rather reflect the opposite direction of regulation under the selected ROC orientation. Therefore, the ROC curves should be interpreted together with the corresponding concentration distributions reported in Fig. S3. Overall, the ROC analysis confirms that LPC species provide the most robust univariate discrimination between HCC and HCV, whereas CARs contribute more modestly and, in some cases, show inverse class associations.

## Discussion

This study aimed to translate, confirm, and validate findings obtained from an untargeted metabolomics and lipidomics approach for the stratification of HCC patients and their discrimination from other chronic liver diseases. Quantitative measurements in metabolomics are pivotal for defining diagnostic thresholds, ensuring inter-study comparability, and enabling clinical implementation. In this context, a targeted method was developed and optimized for the specific analyte panel, including tailored sample preparation and LC–MS conditions to maximize analytical sensitivity and robustness. The method was validated in accordance with established bioanalytical guidelines, supporting its suitability for quantitative metabolomics applications and potential clinical translation.

Concerning sample preparation, the comparative evaluation of SPE and solvent-based precipitation revealed an inherent trade-off between matrix clean-up and the extent of chemical coverage, a phenomenon that is frequently observed in SPE-based methods. While SPE offers selective enrichment and reduced matrix complexity, its performance was class-dependent, which limited the simultaneous recovery of carnitines and LPCs. The findings reported here are consistent with the literature, which suggests that polymeric reversed-phase SPE may preferentially retain or lose analytes depending on polarity and functional group composition, particularly when a single protocol is applied to heterogeneous metabolite classes [27].

Conversely, protein precipitation with ice-cold ACN provided a pragmatic solution for the targeted quantification of carnitines and LPCs, enhancing also reproducibility, as previously outlined in the context of metabolomics workflows [28]. Furthermore, coupled with HILIC downstream separation, ACN precipitation resulted in a substantial enhancement of chromatographic performance, as evidenced by the observation of more defined and symmetrical peaks in comparison to those achieved through MeOH-based precipitation. This observation is in accordance with the extant literature, which suggests that ACN precipitation leads to a reduction in residual protein content and matrix complexity. Polson et al. [29] demonstrated that ACN forms larger protein pellets and drastically reduces residual protein in the supernatant, resulting in cleaner extracts and improved peak quality for LC–MS/MS analysis [30], resulting in higher average recovery and low matrix effect. These results are in line with best-practice guidelines for bioanalytical method validation (ICH M10), which emphasize accuracy, precision, and matrix effect evaluation for clinical translation.

On the chromatographic side, the selection of a BEH Amide HILIC stationary phase was driven by our previous untargeted analyses as well as different analogue studies [31, 32]. Mobile phase composition plays a critical role in HILIC separations, especially with respect to buffering capacity and ionic strength. While acidic mobile phases containing formic acid are frequently employed due to their compatibility with ESI–MS, several studies have demonstrated that volatile ammonium salts, such as ammonium acetate or ammonium formate, can significantly improve peak shape and retention reproducibility by stabilizing the water-rich layer on the stationary phase and modulating electrostatic interactions [33]. In this study, buffered mobile phases containing ammonium acetate outperformed acidic systems, likely due to improved control of ion–stationary phase interactions and reduced peak asymmetry. Finally, the observed chromatographic peak splitting when using an ACN 70% resuspension solvent is consistent with known HILIC injection effects, where mismatches between sample solvent strength and initial mobile phase composition can lead to poor analyte focusing. Increasing the organic content to ACN 80% enhanced on-column focusing and eliminated peak distortion, in agreement with established HILIC best practices [34].

Targeted LC-HRMS assays for CARs and LPCs must cope with broad dynamic ranges, low-abundance features, isobaric/isotopic complexity and ion suppression. In quadrupole-Orbitrap MS analyzers, selected ion monitoring (SIM) offers a practical sensitivity gain versus full scan (FS) by enriching specific *m/z* windows in the Orbitrap analyzer while retaining high-mass accuracy—an approach well suited to complex matrices and large analyte panels. Multiplexing SIM windows (tSIM-MPX) further increases throughput without the need for extensive transition selection typical of triple-quadrupole SRM workflows [35]. Additionally, this approach offers a high number of data points per chromatographic peak, improving peak integration stability and lowering CV% [36]. While SRM/MRM remains a gold standard on triple quadrupoles, the tSIM-MPX approach offers SRM comparable sensitivity with the enhanced selectivity at high resolution, simplified method development, and strong quantitative performance in complex matrices. Compared to traditional SIM, tSIM-MPX monitors multiple targets within a single acquisition event, reducing cycle time while maintaining sensitivity and quantitative accuracy when properly tuned. In fact, a known challenge of trapping analyzers is represented by space-charge effects, which were mitigated by optimizing AGC targets and maximum injection time [37].

Importantly, for lipid classes susceptible to positional isomerism, such as LPCs, chromatographic separation serves as an essential complement to high-resolution/accurate mass (HR/AM) detection. Consistent with established behavior of lysophospholipids [38, 39], informative HCD-MS/MS spectra demonstrated a higher relative abundance of fragment ions corresponding to cleavage of the fatty acyl chain from the *sn-2* position, thereby providing an additional structural descriptor for regioisomer assignment, an additional benefit with respect to limited information provided by quantifier/qualifier ions used in SRM/MRM methods.

The circulating signature showed elevation of short- and long-chain CARs in HCC, which supports the hypothesis of mitochondrial β-oxidation dysregulation, a well-recognized hallmark of cancer metabolism. Previous studies have linked altered CAR profiles to impaired fatty acid oxidation and increased energy demand in rapidly proliferating tumor cells, reinforcing their potential as diagnostic biomarkers for HCC [40]. Conversely, the enrichment of medium-chain CARs in HCV samples suggests a distinct metabolic phenotype associated with chronic viral infection rather than malignant transformation, highlighting the discriminatory power of CAR profiling in differentiating disease states.

The most significant finding is represented by the pronounced reduction in LPCs, which could reflect accelerated membrane remodeling and altered phospholipid turnover, processes essential for sustaining tumor growth and metastasis. This lipidomic signature aligns with previous reports implicating LPC depletion and LPCAT-mediated reacylation in HCC progression [40, 41]. Notably, LPCs represent the strongest discriminative analytes in the measured panel.

A clear limitation of this study lies in the relatively small and unbalanced cohort size. In this context our results should be validated in a larger, prospective, and independent validation cohort, also considering potential differences in demographic and clinical characteristics. Although the influence of clinical and biochemical variables was previously evaluated and no significant correlation with the metabolic signature was observed [12], the relatively small and unbalanced cohort size limits the possibility of fully excluding residual confounding effects. Therefore, future studies in larger, prospective, and independent cohorts will be necessary to validate the robustness of the targeted panel and to more comprehensively assess the impact of demographic and clinical variables.

An additional limitation is the absence of healthy controls, which could be useful to further assess biomarker specificity and the potential use of the panel as a non-invasive liquid biopsy assay for surveillance of individuals with risk factors for HCC. In this regard it should be considered that HCC tends to arise in an already chronically damaged liver; therefore, a diagnostic assay would be more clinically relevant for discriminating patients with HCC from patients with chronic liver disease without HCC, rather than from healthy individuals. This is consistent with the design of the present study, in which HCC patients were compared with HCV patients, a clinically relevant control group where AFP serum levels may be only modestly increased, potentially resulting in false-negative classifications. Nevertheless, the method showed robust performance, matching that5 of the previous untargeted approach, which corroborates its analytical value in future applications, particularly in AFP-negative cases, representing a useful tool for clinical decisions.

## Conclusions

In this study, we established and validated a targeted workflow that enables the quantification of potentially actionable biomarkers for hepatocellular carcinoma (HCC). The rapid, analytically robust tSIM-MPX-based HILIC-HRMS assay successfully confirmed disease-associated molecular signatures and obtained absolute quantitative measurements suitable for clinical interpretation. These results underscore the potential of this targeted panel as a non-invasive liquid biopsy tool for HCC, particularly in AFP-negative cases where conventional biomarkers fail.

## Supplementary Information

Below is the link to the electronic supplementary material.

Supplementary file1 (DOCX 1.03 MB)

## Data Availability

All data are included in the paper. RAW MS files associated are available at Zenodo (https://zenodo.org/) under the following: 10.5281/zenodo.19352347.
