# Supplementary material for: Development and validation of a multiplexed targeted HILIC-HRMS assay for quantitative analysis of hepatocellular carcinoma circulating biomarkers
Source: Anal Bioanal Chem. 2026 May 23;418(14):4305–20. doi: 10.1007/s00216-026-06568-1 (PMC13375758; doi:10.1007/s00216-026-06568-1)
Supplement: Supplementary file 1 — Supplementary file1 (DOCX 1.03 MB) [file 216_2026_6568_MOESM1_ESM.docx]

**Supporting information for the manuscript:**

**Development and validation of a multiplexed targeted HILIC-HRMS assay for quantitative analysis of Hepatocellular Carcinoma circulating biomarkers**

Danila La Gioia^1*^, Vicky Caponigro^1*^, Anna Lucia Tornesello^2^, Emanuela Salviati^1^, Antonio Malinconico^1, 4^, Fabrizio Merciai^1^, Luigi Buonaguro^2^, Franco M Buonaguro^3^, Pietro Campiglia^1^, Maria Lina Tornesello^3^, Eduardo Sommella^1#^

^1^Department of Pharmacy, University of Salerno, Via Giovanni Paolo II 132, Fisciano, SA, Italy.

^2^Innovative Immunological Models Unit, Istituto Nazionale Tumori IRCCS "Fondazione G. Pascale", 80131, Naples, Italy.

^3^Molecular Biology and Viral Oncology Unit, Istituto Nazionale Tumori IRCCS "Fondazione G. Pascale", 80131, Naples, Italy.

^4^ National PhD Program in "RNA Therapeutics and Gene Therapy", Napoli, Italy

* These authors share co-first authorship

# Corresponding author

**S1. Participants’ characteristics and collection of clinical samples**

All HCC patients were in BCLC stage A or stage B and treated by surgical liver resection according to Milan criteria. Chronic HCV infection was defined, in agreement with the Centre for Disease Controls and Prevention (CDC) guidelines, as a persistent viremia occurring for greater than 6 months after the initial exposure. The HCV infection was diagnosed by detection of anti-HCV antibodies with third-generation enzyme immunoassay (III generation EIA) against HCV-core and HCV-non-structural antigens and confirmed by detection of HCV RNA (Cobas Amplicor HCV assay, ROCHE). For each patient, the age at diagnosis, gender, HCV status, HCV viral load when available, and diagnosis were recorded. Liver function tests, including Alphafetoprotein (AFP), Carbohydrate antigen 19-9 (CA19-9), Carcinoembryonic antigen (CEA), Alanine aminotransferase (ALT), Aspartate Aminotransferase (AST) and Gamma-glutamyl transferase (GGT), have been also retrospectively collected. Serological testing for tumor biomarkers was carried out with regulatory agencies–approved and commercially available kits according to the manufacturers’ instructions. The upper limits of tumor biomarkers standard reference values were AFP ≤ 20 ng/L, CEA ≤ 3 ng/L, and CA19-9 ≤ 37 U/m. Blood samples were obtained from all HCV-positive subjects before the initiation of any type of treatment including the use of direct acting antivirals. For each patient, 5 mL of whole blood was collected in ethylenediaminetetraacetic acid (EDTA) tubes and processed within 2 h after collection. The plasma samples were obtained by centrifugation at 1200×g for 15 min and then stored at − 80 °C. HCC patients were classified according to ChildPugh score into A (n = 40) and B (n = 8). Tumor size and the number of tumor nodules were determined by computed tomography or magnetic resonance imaging. HCC were classified in moderately differentiated (G2 n = 46) and poorly differentiated (G3 n = 2) tumors, according to the histological grade criteria defined by Edmondson and Steiner [19]. The study was approved by the Institutional Scientific Board and by the Ethical Committee of the Istituto Nazionale Tumori “Fondazione G. Pascale” (prot. 51-OSS/21), and it is in accordance with the principles of the Declaration of Helsinki.

**S2. Solid Phase Extraction Protocol**

**Pre-treatment**

Plasma samples were extracted as follow:: 30 µL of plasma were thawed on ice and 300 µL of ice-cold ACN, containing a mix of deuterated standards (C16-Carnitine, C8-Carnitine, C5-Carnitine, LPC 17:0, LPC 18:1, C16 Carnitine-d9, C8 Carnitine-d9, C5 Carnitine-d9, LPC 15:0-d5, LPC 17:0-d5, LPC 19:0-d5) were added and vortexed for 10 min. Subsequently, samples were incubated at -30 °C for 30 min and centrifuged at 14,680 rpm, for 10 min at 4 °C to induce protein precipitation.

The supernatant was collected and kept on ice prior to solid-phase extraction (SPE), which was performed using Strata-X polymeric reversed-phase cartridges (10 mg, 1 mL, 33 µm; Phenomenex, Torrance, CA, USA).

Cartridges were conditioned with 1 mL of methanol followed by equilibration with 1 mL of water. Subsequently, 300 µL of the previously collected supernatant were loaded onto the cartridge. Different washing solutions (1 mL of 5% MeOH (v/v), 1 mL of 5% ACN (v/v), or 1 mL of 2% ACN (v/v)) and elution solvents (1 mL of MeOH + 0.1% formic acid or ACN + 0.1% formic acid) were tested. However, this protocol did not yield satisfactory recovery results (Table **S1**).

| **Washing (W) and Elution (E) solutions** | **Recovery_avg_** | |
| --- | --- | --- |
|  | **CARs** | **LPCs** |
| W: MeOH 5% (*v/v*), E: MeOH + 0.1% HCOOH | 27.42% | 62.25% |
| W: MeOH 5% (*v/v*), E: ACN + 0.1% HCOOH | 17.25% | 57.45% |
| W: ACN 5% (*v/v*), E: MeOH + 0.1% HCOOH | 22.78% | 67.87% |
| W: ACN 5% (*v/v*), E: ACN + 0.1% HCOOH | 15.98% | 62.45% |
| W: ACN 2% (*v/v*), E: MeOH + 0.1% HCOOH | 32.54% | 72.21% |
| W: ACN 2% (*v/v*), E: ACN + 0.1% HCOOH | 25.28% | 75.23% |

**Tab S1**. Recovery of carnitines (CARs) and lysophosphatidylcholines (LPCs) obtained using the SPE protocol under different washing and elution conditions.


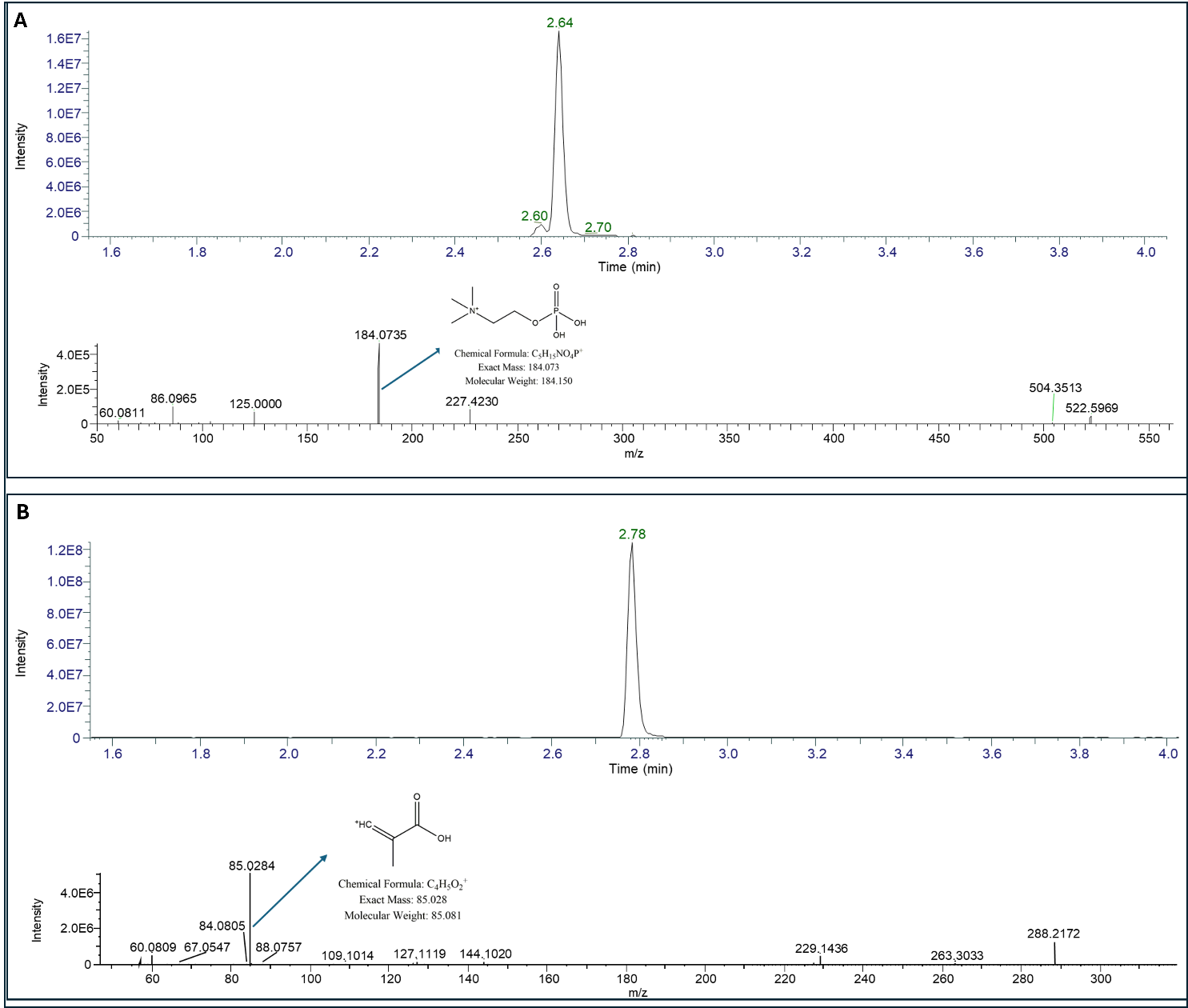


**Fig**. **S1 A-B** Targeted MS/MS spectra of LPCs and CARs. A. LPCs were confirmed by the diagnostic phosphocholine fragment at m/z 184.0733 (C₅H₁₅NO₄P⁺), while CARs were validated by the characteristic fragment at m/z 85.0284 (C₄H₅O₂⁺).

**
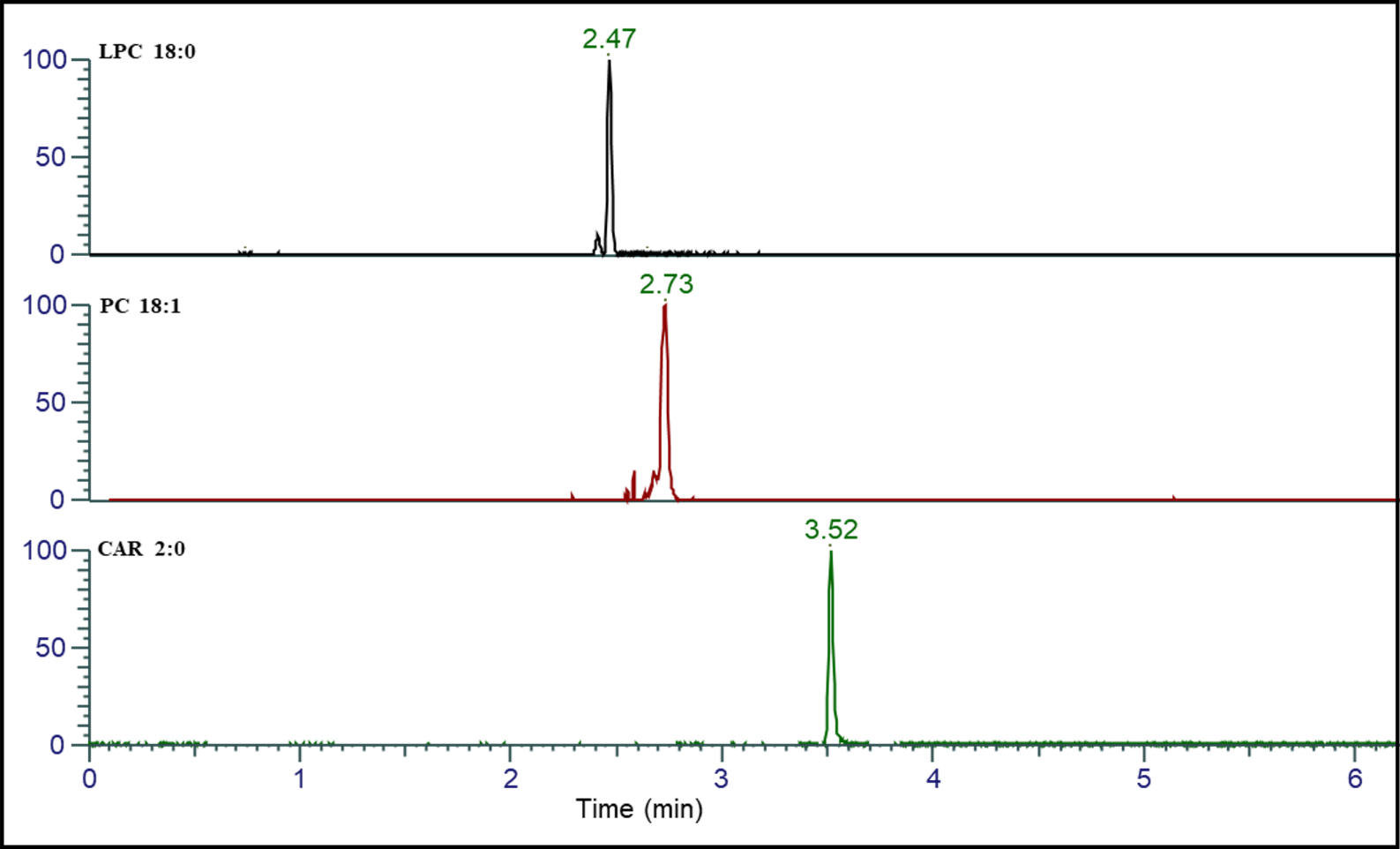
**

**Fig. S2** Elution window for LPC 18:0, PC 18:1 and CAR C2:0 detected in human plasma.

**
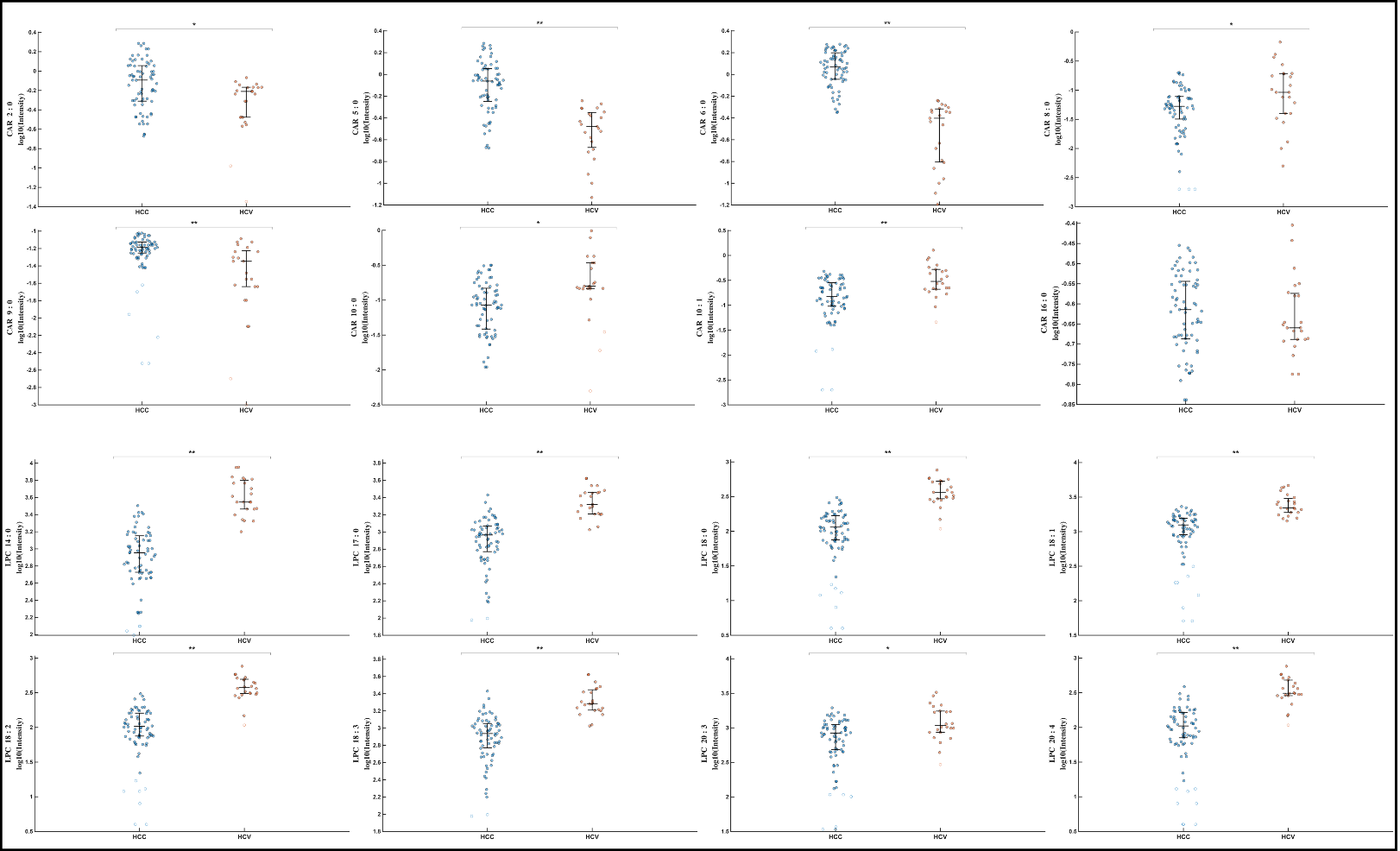
**

**Fig. S3** Scatter plots for individual metabolites report log10 transformed concentration values. Statistical differences between HCC and HCV groups were assessed using the non-parametric Mann - Whitney U test followed by FDR correction. * pvalue < 0.05; ** pvalue < 0.001

|  | **CAR 10:0** | **CAR 10:1** | **CAR 16:0** | **CAR 2:0** | **CAR 5:0** | **CAR 6:0** | **CAR 8:0** | **CAR 9:0** | **LPC 14:0** | **LPC 17:0** | **LPC 18:0** | **LPC 18:1** | **LPC 18:2** | **LPC 18:3** | **LPC 20:3** | **LPC 20:4** |
| --- | --- | --- | --- | --- | --- | --- | --- | --- | --- | --- | --- | --- | --- | --- | --- | --- |
| **HCC** | 0,011 | 0,002 | 0,145 | 0,285 | 0,354 | 1,102 | 0,135 | 0,069 | 2,664 | 1,485 | 0,143 | 0,051 | 0,143 | 1,485 | 0,034 | 0,143 |
|  | 0,023 | 0,042 | 0,169 | 0,223 | 0,211 | 0,926 | 0,054 | 0,06 | 0,125 | 0,982 | 0,015 | 0,183 | 0,012 | 1,486 | 0,108 | 0,004 |
|  | 0,032 | 0,046 | 0,176 | 0,336 | 0,213 | 1,74 | 0,073 | 0,062 | 0,182 | 1,217 | 0,004 | 0,337 | 0,004 | 1,138 | 0,168 | 0,013 |
|  | 0,032 | 0,069 | 0,192 | 0,365 | 0,223 | 1,381 | 0,103 | 0,038 | 0,446 | 0,949 | 0,004 | 0,601 | 0,004 | 0,982 | 0,286 | 0,038 |
|  | 0,043 | 0,095 | 0,208 | 0,496 | 0,285 | 0,773 | 0,071 | 0,003 | 0,521 | 0,777 | 0,008 | 0,847 | 0,008 | 1,013 | 0,454 | 0,059 |
|  | 0,053 | 0,104 | 0,214 | 0,519 | 0,304 | 1,578 | 0,012 | 0,072 | 0,534 | 0,48 | 0,012 | 0,884 | 0,012 | 0,834 | 0,483 | 0,062 |
|  | 0,011 | 0,002 | 0,145 | 0,285 | 0,336 | 0,682 | 0,135 | 0,069 | 2,664 | 1,485 | 0,013 | 0,051 | 0,013 | 1,217 | 0,034 | 0,143 |
|  | 0,013 | 0,012 | 0,162 | 0,336 | 0,341 | 1,228 | 0,138 | 0,062 | 0,098 | 1,486 | 0,017 | 0,079 | 0,017 | 0,679 | 0,037 | 0,008 |
|  | 0,015 | 0,013 | 0,169 | 0,213 | 0,355 | 1,315 | 0,004 | 0,075 | 0,11 | 1,138 | 0,022 | 0,121 | 0,022 | 0,625 | 0,101 | 0,004 |
|  | 0,023 | 0,048 | 0,168 | 0,223 | 0,365 | 1,897 | 0,054 | 0,06 | 0,125 | 0,982 | 0,038 | 0,183 | 0,038 | 0,949 | 0,108 | 0,004 |
|  | 0,028 | 0,044 | 0,171 | 0,285 | 0,447 | 0,859 | 0,078 | 0,063 | 0,176 | 1,013 | 0,042 | 0,226 | 0,042 | 1,125 | 0,132 | 0,008 |
|  | 0,029 | 0,044 | 0,172 | 0,304 | 0,451 | 1,438 | 0,189 | 0,048 | 0,182 | 0,834 | 0,055 | 0,314 | 0,055 | 0,815 | 0,136 | 0,012 |
|  | 0,032 | 0,046 | 0,176 | 0,336 | 0,488 | 0,694 | 0,073 | 0,062 | 0,182 | 1,217 | 0,057 | 0,337 | 0,057 | 1,052 | 0,168 | 0,013 |
|  | 0,035 | 0,047 | 0,178 | 0,341 | 0,495 | 1,001 | 0,045 | 0,095 | 0,253 | 0,679 | 0,057 | 0,43 | 0,057 | 1,185 | 0,228 | 0,017 |
|  | 0,037 | 0,061 | 0,194 | 0,355 | 0,496 | 1,842 | 0,008 | 0,095 | 0,391 | 0,625 | 0,059 | 0,489 | 0,059 | 0,777 | 0,279 | 0,022 |
|  | 0,032 | 0,069 | 0,192 | 0,365 | 0,517 | 0,533 | 0,103 | 0,038 | 0,446 | 0,949 | 0,059 | 0,601 | 0,059 | 0,667 | 0,286 | 0,038 |
|  | 0,032 | 0,071 | 0,199 | 0,447 | 0,519 | 1,682 | 0,032 | 0,065 | 0,45 | 1,125 | 0,062 | 0,701 | 0,062 | 0,48 | 0,379 | 0,042 |
|  | 0,034 | 0,083 | 0,201 | 0,451 | 0,582 | 1,212 | 0,065 | 0,071 | 0,458 | 0,815 | 0,068 | 0,702 | 0,068 | 0,736 | 0,399 | 0,055 |
|  | 0,037 | 0,085 | 0,204 | 0,488 | 0,583 | 1,064 | 0,054 | 0,064 | 0,458 | 1,052 | 0,071 | 0,726 | 0,071 | 1,116 | 0,443 | 0,057 |
|  | 0,039 | 0,088 | 0,206 | 0,495 | 0,611 | 0,91 | 0,078 | 0,063 | 0,464 | 1,185 | 0,071 | 0,841 | 0,071 | 0,922 | 0,45 | 0,057 |
|  | 0,043 | 0,095 | 0,208 | 0,496 | 0,62 | 1,336 | 0,071 | 0,003 | 0,521 | 0,777 | 0,073 | 0,847 | 0,073 | 0,613 | 0,45 | 0,059 |
|  | 0,051 | 0,096 | 0,212 | 0,517 | 0,623 | 0,481 | 0,051 | 0,065 | 0,524 | 0,667 | 0,074 | 0,883 | 0,074 | 1,231 | 0,451 | 0,059 |
|  | 0,053 | 0,104 | 0,213 | 0,519 | 0,636 | 0,758 | 0,012 | 0,072 | 0,534 | 0,48 | 0,075 | 0,884 | 0,075 | 1,454 | 0,483 | 0,062 |
|  | 0,062 | 0,104 | 0,278 | 0,582 | 0,645 | 1,742 | 0,009 | 0,084 | 0,548 | 0,736 | 0,075 | 0,912 | 0,075 | 1,543 | 0,506 | 0,068 |
|  | 0,073 | 0,104 | 0,217 | 0,583 | 0,656 | 1,652 | 0,02 | 0,075 | 0,552 | 1,116 | 0,076 | 0,933 | 0,076 | 0,497 | 0,556 | 0,071 |
|  | 0,074 | 0,107 | 0,224 | 0,611 | 0,713 | 1,489 | 0,025 | 0,058 | 0,563 | 0,922 | 0,077 | 0,979 | 0,077 | 1,056 | 0,621 | 0,071 |
|  | 0,076 | 0,113 | 0,226 | 0,62 | 0,744 | 0,889 | 0,048 | 0,04 | 0,563 | 0,613 | 0,079 | 0,999 | 0,079 | 1,061 | 0,637 | 0,073 |
|  | 0,076 | 0,119 | 0,226 | 0,623 | 0,804 | 1,155 | 0,064 | 0,054 | 0,598 | 1,231 | 0,082 | 1,008 | 0,082 | 0,941 | 0,65 | 0,074 |
|  | 0,078 | 0,12 | 0,227 | 0,636 | 0,807 | 1,805 | 0,143 | 0,011 | 0,614 | 1,454 | 0,088 | 1,056 | 0,088 | 1,087 | 0,676 | 0,075 |
|  | 0,079 | 0,122 | 0,23 | 0,645 | 0,825 | 0,902 | 0,184 | 0,056 | 0,642 | 1,543 | 0,093 | 1,08 | 0,093 | 1,339 | 0,689 | 0,075 |
|  | 0,084 | 0,124 | 0,239 | 0,657 | 0,869 | 1,373 | 0,12 | 0,038 | 0,661 | 0,497 | 0,094 | 1,092 | 0,094 | 0,932 | 0,735 | 0,076 |
|  | 0,085 | 0,132 | 0,24 | 0,713 | 0,873 | 1,293 | 0,022 | 0,055 | 0,683 | 1,056 | 0,098 | 1,092 | 0,098 | 0,699 | 0,752 | 0,077 |
|  | 0,085 | 0,137 | 0,241 | 0,744 | 0,884 | 1,741 | 0,101 | 0,024 | 0,704 | 1,061 | 0,099 | 1,139 | 0,099 | 1,571 | 0,752 | 0,079 |
|  | 0,094 | 0,144 | 0,243 | 0,804 | 0,884 | 0,567 | 0,039 | 0,05 | 0,75 | 0,941 | 0,101 | 1,163 | 0,101 | 0,888 | 0,791 | 0,082 |
|  | 0,089 | 0,141 | 0,249 | 0,807 | 0,884 | 1,786 | 0,035 | 0,042 | 0,787 | 1,087 | 0,102 | 1,167 | 0,102 | 0,865 | 0,847 | 0,088 |
|  | 0,094 | 0,143 | 0,25 | 0,825 | 0,896 | 1,606 | 0,02 | 0,049 | 0,819 | 1,339 | 0,105 | 1,191 | 0,105 | 0,515 | 0,806 | 0,093 |
|  | 0,096 | 0,15 | 0,252 | 0,869 | 0,915 | 0,986 | 0,023 | 0,075 | 0,862 | 0,932 | 0,111 | 1,219 | 0,111 | 0,703 | 0,822 | 0,094 |
|  | 0,103 | 0,162 | 0,254 | 0,873 | 0,919 | 1,443 | 0,076 | 0,006 | 0,867 | 0,699 | 0,113 | 1,237 | 0,113 | 0,662 | 0,842 | 0,098 |
|  | 0,104 | 0,183 | 0,257 | 0,884 | 0,923 | 0,845 | 0,109 | 0,064 | 0,901 | 1,571 | 0,113 | 1,253 | 0,113 | 2,206 | 0,849 | 0,099 |
|  | 0,108 | 0,192 | 0,259 | 0,884 | 0,943 | 1,172 | 0,05 | 0,049 | 0,908 | 0,888 | 0,116 | 1,305 | 0,116 | 0,466 | 0,853 | 0,101 |
|  | 0,111 | 0,206 | 0,261 | 0,896 | 0,986 | 0,941 | 0,076 | 0,052 | 0,944 | 0,515 | 0,12 | 1,37 | 0,12 | 1,853 | 0,918 | 0,105 |
|  | 0,116 | 0,206 | 0,271 | 0,915 | 0,99 | 1,593 | 0,049 | 0,05 | 0,955 | 0,703 | 0,129 | 1,38 | 0,129 | 0,878 | 0,922 | 0,111 |
|  | 0,117 | 0,221 | 0,276 | 0,919 | 0,996 | 1,128 | 0,002 | 0,069 | 0,973 | 0,662 | 0,129 | 1,383 | 0,129 | 1,744 | 0,951 | 0,113 |
|  | 0,119 | 0,223 | 0,28 | 0,923 | 1,005 | 0,631 | 0,057 | 0,069 | 1,005 | 2,206 | 0,131 | 1,389 | 0,131 | 1,222 | 0,964 | 0,113 |
|  | 0,128 | 0,223 | 0,28 | 0,943 | 1,126 | 1,663 | 0,041 | 0,073 | 1,008 | 0,466 | 0,133 | 1,442 | 0,133 | 2,686 | 0,985 | 0,116 |
|  | 0,129 | 0,225 | 0,28 | 0,985 | 1,127 | 1,768 | 0,201 | 0,02 | 1,041 | 1,378 | 0,142 | 1,443 | 0,142 | 0,939 | 0,995 | 0,12 |
|  | 0,134 | 0,225 | 0,282 | 0,986 | 1,138 | 1,049 | 0,016 | 0,083 | 1,068 | 1,853 | 0,159 | 1,453 | 0,159 | 0,678 | 0,998 | 0,12 |
|  | 0,138 | 0,228 | 0,288 | 0,997 | 1,138 | 1,641 | 0,097 | 0,056 | 1,068 | 0,878 | 0,161 | 1,455 | 0,161 | 1,297 | 1,004 | 0,129 |
|  | 0,145 | 0,243 | 0,295 | 0,996 | 1,149 | 1,397 | 0,018 | 0,076 | 1,085 | 1,744 | 0,162 | 1,463 | 0,162 | 0,763 | 1,014 | 0,129 |
|  | 0,157 | 0,257 | 0,303 | 1,124 | 1,234 | 1,146 | 0,199 | 0,061 | 1,22 | 2,686 | 0,169 | 1,474 | 0,169 | 1,071 | 1,033 | 0,133 |
|  | 0,159 | 0,259 | 0,304 | 1,127 | 1,267 | 1,308 | 0,121 | 0,055 | 1,248 | 0,939 | 0,173 | 1,511 | 0,173 | 0,263 | 1,041 | 0,142 |
|  | 0,165 | 0,268 | 0,305 | 1,138 | 1,314 | 0,453 | 0,035 | 0,059 | 1,257 | 0,678 | 0,174 | 1,518 | 0,174 | 0,369 | 1,068 | 0,159 |
|  | 0,178 | 0,278 | 0,307 | 1,138 | 1,33 | 0,985 | 0,056 | 0,067 | 1,266 | 1,297 | 0,178 | 1,532 | 0,178 | 0,462 | 1,113 | 0,161 |
|  | 0,199 | 0,297 | 0,313 | 1,149 | 1,449 | 1,052 | 0,048 | 0,039 | 1,317 | 0,763 | 0,178 | 1,539 | 0,178 | 0,498 | 1,122 | 0,162 |
|  | 0,213 | 0,339 | 0,318 | 1,234 | 1,461 | 1,373 | 0,052 | 0,086 | 1,42 | 1,071 | 0,182 | 1,649 | 0,182 | 0,309 | 1,173 | 0,169 |
|  | 0,212 | 0,341 | 0,32 | 1,267 | 1,696 | 1,135 | 0,019 | 0,063 | 1,43 | 0,263 | 0,194 | 1,666 | 0,194 | 0,583 | 1,244 | 0,173 |
|  | 0,246 | 0,351 | 0,327 | 1,314 | 1,751 | 0,605 | 0,012 | 0,079 | 1,47 | 0,369 | 0,195 | 1,676 | 0,195 | 0,824 | 1,256 | 0,174 |
|  | 0,246 | 0,363 | 0,351 | 1,334 | 1,827 | 0,764 | 0,058 | 0,072 | 1,533 | 0,462 | 0,197 | 1,709 | 0,197 | 0,276 | 1,266 | 0,178 |
|  | 0,265 | 0,372 | 0,28 | 1,449 | 1,929 | 1,887 | 0,016 | 0,089 | 1,538 | 0,498 | 0,252 | 1,735 | 0,252 | 0,174 | 1,288 | 0,178 |
|  | 0,272 | 0,402 | 0,224 | 1,454 | 1,732 | 1,487 | 0,058 | 0,089 | 1,559 | 0,432 | 0,257 | 1,771 | 0,257 | 0,194 | 1,296 | 0,179 |
|  | 0,314 | 0,412 | 0,285 | 1,461 | 1,105 | 0,902 | 0,002 | 0,069 | 1,575 | 0,309 | 0,282 | 1,777 | 0,282 | 0,099 | 1,31 | 0,182 |
|  | 0,316 | 0,48 | 0,318 | 1,696 | 0,846 | 1,264 | 0,05 | 0,069 | 1,589 | 0,583 | 0,306 | 1,78 | 0,306 | 0,158 | 1,325 | 0,194 |
|  | 0,076 | 0,402 | 0,306 | 1,827 | 1,21 | 1,798 | 0,045 | 0,088 | 1,782 | 0,276 | 0,168 | 1,849 | 0,088 | 0,095 | 1,474 | 0,197 |
|  | 0,085 | 0,352 | 0,295 | 1,929 | 0,975 | 1,039 | 0,015 | 0,07 | 1,807 | 0,174 | 0,242 | 1,937 | 0,098 | 0,421 | 1,528 | 0,252 |
|  | 0,092 | 0,382 | 0,278 | 1,187 | 1,845 | 0,588 | 0,041 | 0,085 | 2,046 | 0,194 | 0,154 | 1,968 | 0,129 | 0,365 | 1,533 | 0,257 |
|  | 0,178 | 0,356 | 0,245 | 1,335 | 0,624 | 1,672 | 0,048 | 0,092 | 2,398 | 0,099 | 0,256 | 2,04 | 0,161 | 1,25 | 1,596 | 0,282 |
|  | 0,212 | 0,417 | 0,341 | 1,696 | 1,563 | 1,12 | 0,032 | 0,092 | 2,414 | 0,158 | 0,139 | 2,068 | 0,197 | 0,704 | 1,675 | 0,306 |
|  | 0,272 | 0,348 | 0,346 | 1,929 | 0,856 | 1,356 | 0,069 | 0,093 | 2,564 | 0,153 | 0,142 | 2,219 | 0,085 | 0,34 | 1,949 | 0,387 |
|  | 0,316 | 0,324 | 0,328 | 1,267 | 0,799 | 0,447 | 0,002 | 0,06 | 3,202 | 0,095 | 0,131 | 2,31 | 0,079 | 0,859 | 1,474 | 0,194 |
| **HCV** | 0,005 | 0,046 | 0,187 | 0,045 | 0,432 | 0,577 | 0,005 | 0,016 | 2,193 | 1,904 | 0,148 | 1,429 | 0,148 | 1,904 | 0,294 | 0,148 |
|  | 0,019 | 0,093 | 0,168 | 0,105 | 0,294 | 0,47 | 0,01 | 0,049 | 1,588 | 1,06 | 0,108 | 1,544 | 0,108 | 1,06 | 0,44 | 0,108 |
|  | 0,103 | 0,187 | 0,203 | 0,296 | 0,487 | 0,064 | 0,033 | 0,058 | 2,49 | 4,181 | 0,268 | 1,747 | 0,268 | 4,181 | 0,72 | 0,268 |
|  | 0,145 | 0,214 | 0,205 | 0,333 | 0,205 | 0,531 | 0,04 | 0,045 | 2,913 | 1,608 | 0,287 | 1,895 | 0,287 | 1,608 | 0,863 | 0,287 |
|  | 0,147 | 0,217 | 0,206 | 0,335 | 0,537 | 0,155 | 0,061 | 0,05 | 2,96 | 3,04 | 0,294 | 2,088 | 0,294 | 3,04 | 0,942 | 0,294 |
|  | 0,147 | 0,223 | 0,215 | 0,489 | 0,493 | 0,524 | 0,076 | 0,028 | 3,509 | 3,449 | 0,385 | 2,132 | 0,348 | 3,449 | 0,988 | 0,345 |
|  | 0,152 | 0,272 | 0,219 | 0,584 | 0,332 | 0,344 | 0,092 | 0,008 | 3,517 | 2,852 | 0,309 | 2,205 | 0,309 | 2,852 | 1,026 | 0,309 |
|  | 0,158 | 0,334 | 0,223 | 0,617 | 0,37 | 0,209 | 0,103 | 0,023 | 4,106 | 4,185 | 0,314 | 2,488 | 0,314 | 4,185 | 1,136 | 0,314 |
|  | 0,172 | 0,387 | 0,226 | 0,617 | 0,167 | 0,456 | 0,121 | 0,075 | 4,397 | 1,639 | 0,364 | 2,524 | 0,364 | 1,639 | 1,154 | 0,364 |
|  | 0,18 | 0,435 | 0,263 | 0,675 | 0,319 | 0,162 | 0,17 | 0,082 | 5,06 | 2,899 | 0,366 | 2,706 | 0,366 | 2,899 | 1,678 | 0,366 |
|  | 0,284 | 0,473 | 0,268 | 0,681 | 0,49 | 0,368 | 0,176 | 0,058 | 5,845 | 2,643 | 0,391 | 2,71 | 0,391 | 2,63 | 1,697 | 0,391 |
|  | 0,342 | 0,499 | 0,279 | 0,721 | 0,115 | 0,419 | 0,19 | 0,016 | 5,855 | 2,084 | 0,435 | 2,776 | 0,435 | 2,084 | 1,72 | 0,435 |
|  | 0,424 | 0,524 | 0,282 | 0,733 | 0,425 | 0,498 | 0,194 | 0,07 | 6,512 | 1,595 | 0,481 | 3,115 | 0,481 | 1,595 | 1,747 | 0,481 |
|  | 0,785 | 0,577 | 0,308 | 0,856 | 0,263 | 0,574 | 0,275 | 0,065 | 6,71 | 2,588 | 0,514 | 3,431 | 0,514 | 2,588 | 2,044 | 0,514 |
|  | 0,981 | 0,643 | 0,361 | 0,627 | 0,573 | 0,081 | 0,368 | 0,023 | 6,914 | 1,437 | 0,526 | 3,933 | 0,526 | 1,437 | 2,137 | 0,526 |
|  | 0,158 | 0,823 | 0,394 | 0,675 | 0,121 | 0,137 | 0,413 | 0,001 | 8,934 | 1,725 | 0,579 | 4,33 | 0,579 | 1,725 | 2,285 | 0,579 |
|  | 0,342 | 0,896 | 0,226 | 0,681 | 0,348 | 0,396 | 0,671 | 0,024 | 8,987 | 2,027 | 0,584 | 4,429 | 0,584 | 2,027 | 2,906 | 0,584 |
|  | 0,424 | 1,279 | 0,263 | 0,779 | 0,074 | 0,487 | 0,194 | 0,075 | 6,512 | 1,963 | 0,763 | 4,651 | 0,763 | 1,963 | 3,265 | 0,763 |
|  | 0,035 | 0,145 | 0,168 | 0,274 | 0,452 | 0,115 | 0,013 | 0,033 | 2,114 | 1,15 | 0,536 | 1,57 | 0,495 | 1,438 | 0,61 | 0,153 |
|  | 0,052 | 0,168 | 0,197 | 0,279 | 0,241 | 0,233 | 0,028 | 0,002 | 2,13 | 2,825 | 0,327 | 1,676 | 0,314 | 1,927 | 0,705 | 0,216 |
|  | 0,145 | 0,21 | 0,205 | 0,333 | 0,3 | 0,448 | 0,04 | 0,045 | 2,913 | 1,608 | 0,219 | 1,895 | 0,384 | 1,713 | 0,863 | 0,287 |
|  | 0,147 | 0,223 | 0,215 | 0,489 | 0,417 | 0,142 | 0,076 | 0,028 | 3,509 | 3,449 | 0,561 | 2,132 | 0,447 | 1,092 | 0,988 | 0,345 |
|  | 0,152 | 0,272 | 0,219 | 0,582 | 0,194 | 0,45 | 0,092 | 0,008 | 3,517 | 2,852 | 0,281 | 2,205 | 0,329 | 1,864 | 1,026 | 0,309 |

**Tab S2.** Plasma levels of carnitines (CARs) and lysophosphatidylcholines (LPCs) in hepatocellular carcinoma (HCC) and hepatitis C virus (HCV) patients. Concentrations are expressed in µM.
